# Impact of Short-Term Treatment with Telmisartan on Cerebral Arterial Remodeling in SHR

**DOI:** 10.1371/journal.pone.0110766

**Published:** 2014-10-15

**Authors:** Sébastien Foulquier, Isabelle Lartaud, François Dupuis

**Affiliations:** Université de Lorraine, CITHÉFOR “Drug targets, formulation and preclinical assessment,” EA3452, Faculté de Pharmacie, Nancy, France; Charité Universitätsmedizin Berlin, Germany

## Abstract

**Background and Purpose:**

Chronic hypertension decreases internal diameter of cerebral arteries and arterioles. We recently showed that short-term treatment with the angiotensin II receptor blocker telmisartan restored baseline internal diameter of small cerebral arterioles in spontaneously hypertensive rats (SHR), via reversal of structural remodeling and inhibition of the angiotensin II vasoconstrictor response. As larger arteries also participate in the regulation of cerebral circulation, we evaluated whether similar short-term treatment affects middle cerebral arteries of SHR.

**Methods:**

Baseline internal diameters of pressurised middle cerebral arteries from SHR and their respective controls, Wistar Kyoto rats (WKY) and responses to angiotensin II were studied in a small vessel arteriograph. Pressure myogenic curves and passive internal diameters were measured following EDTA deactivation, and elastic modulus from stress-strain relationships.

**Results:**

Active baseline internal diameter was 23% lower in SHR compared to WKY, passive internal diameter (EDTA) 28% lower and elastic modulus unchanged. Pressure myogenic curves were shifted to higher pressure values in SHR. Telmisartan lowered blood pressure but had no effect on baseline internal diameter nor on structural remodeling (passive internal diameter and elastic modulus remained unchanged compared to SHR). Telmisartan shifted the pressure myogenic curve to lower pressure values than SHR.

**Conclusion:**

In the middle cerebral arteries of SHR, short-term treatment with telmisartan had no effect on structural remodeling and did not restore baseline internal diameter, but allowed myogenic tone to adapt towards lower pressure values.

## Introduction

Chronic hypertension decreases internal diameter (ID) of cerebral arterioles [1]–[3]. This decrease in ID is due to changes in structure and/or function of the arterioles. This contributes to the increase in cerebrovascular resistance and to the rightward shift of the limits of cerebral blood flow autoregulation, protecting the brain against higher blood pressure during hypertension [4], [5].

However, at the onset of an antihypertensive treatment, such rightward shift of the lower limit of cerebral blood flow autoregulation - if not early reversed - may be responsible for iatrogenic cerebral hypoperfusion and neuronal dysfunction during the onset decrease in blood pressure. Therefore, it is beneficial for an antihypertensive drug to be accompanied by early reversal of remodeling. Studies at the onset of treatment are scarce. Angiotensin converting enzyme inhibitors (ACEI) early reset the lower limit of cerebral blood flow autoregulation in hypertensives [4], [6], and protect against ischemia [7], but this has been less studied using angiotensin II receptor blockers (ARB). One study showed a first functional remodeling of cerebral circulation in SHR only after 7 to 14 days of candesartan treatment [8]. Similarly, in the SCAST study [9], 7 days with candesartan did not improve post-stroke outcome of hypertensive patients. Therefore it is highly needed to identify anti-hypertensive drugs able to protect the cerebrovasculature soon after treatment initiation. This may be of relevance for early stroke management and stroke outcome in humans.

As we previously showed that 10 days treatment with telmisartan (TELMI) - but not candesartan - reverses the narrowing of pial arteriolar ID in spontaneously hypertensive rats (SHR), *via* reversal of structural remodeling and inhibition of the angiotensin II (AngII) vasoconstrictor response [10], we therefore further investigated the early therapeutic impact of TELMI on cerebral circulation.

Cerebral circulation is regulated at different levels and intracranial arteries upstream of the cerebral arterioles play a major role in cerebrovascular resistance [11], [12]. To further investigate the effect of a short term treatment with TELMI on cerebral circulation, we studied its impact on cerebral macrocirculation. The objective of our study was to evaluate the ability of a short-term treatment with TELMI to reverse the hypertension-induced remodeling of the middle cerebral artery (MCA). For this purpose, structural and mechanical properties of MCA were evaluated in hypertensive rats after a 10-day treatment with TELMI.

## Methods

### Animals and treatments

All experiments were performed in accordance with the European Community guidelines (2010/63/EU) for the use of experimental animals and the respect of the 3 Rs’ requirements for Animal Welfare. Animals were kept under standard conditions (temperature: 21±1°C, hygrometry 60±10%, light on 6 am to 6 pm) and had free access to standard diet (A04, Safe, Villemoisson-sur-Orge, France) and water (reverse osmosis system, Culligan, Brussells, Belgium). The present study and the corresponding treatments of the animals have been performed before February 8, 2013, starting date for the application of the european directive 2010/63/EU in France. The procedures were similar to those described in our previous study [10], in our personal agreements and in a project approved by the regional ethic committee (CELMEA, agreement number CELMEA-2013-0008). The personal permit numbers for animal experimentation (I. Lartaud permit number n° 54-5, F. Dupuis n° 54-105) have been approved by the French Ministry of Agriculture, Paris, France.

Before being sacrificed, animals received orally by daily gavage the treatments or vehicle solutions described below (next paragraph). The rats were then anaesthetized with isoflurane to perform an injection of sodium pentobarbitone and heparin. After the establishment of the anaesthesia, a catheter was placed in the left femoral artery to measure systemic arterial blood pressure. The animals were then decapitated and the brains (together with the middle cerebral arteries) were extracted from the cranial box. The investigator was blinded to the treatment and at early stage of data analysis. The experimental groups were revealed only at the end for final statistical analysis.

The rats used in the present study had the same age, and TELMI was given orally at the same dose (2 mg/kg/day) and duration (10 days) than in our previous study on small pial arterioles [10]. In total 58 rats were used in this study. The experiments were conducted on 4 to 5-month-old male SHR and normotensive Wistar-Kyoto rats (Janvier, Le Genest-Saint-Isles, France). SHR were treated for 10 days by TELMI (2 mg/kg per day, group SHR + TELMI, n = 17 in total) dissolved in drinking water containing mannitol 8.10^−4 ^mol/L + NaOH 6.10^−5 ^mol/L [2], [3], [10]. Control SHR (group SHR, n = 21 in total) and WKY (group WKY, n = 20 in total) received drinking water with solvents for TELMI. Fluid intake and body weight were recorded twice a week in order to adapt concentrations of TELMI and its solvents. WKY rats were heavier than the SHR at the same age. This classic feature has been observed in WKY/SHR rats from different breeding sources. At the end of the treatment period, WKY weighed 510±21 g, SHR 372±4 g and SHR + TELMI 361±2 g. None of the treatments had any impact on animals’ weight and there was no dropout of animals following the treatments.

### Preparation of the middle cerebral artery

The rats were anaesthetized with isoflurane (4% in O_2_) for 5 minutes, then received sodium pentobarbitone (60 mg.kg^−1^, i.p.), and heparin (1000 IU.kg^−1^, i.v. penis vein). A catheter was placed in the left femoral artery to measure systemic arterial mean blood pressure (MAP). Next, the brain was removed from the skull and placed in a physiological salt solution (PSS) at 4°C (mmol.L^−1^: NaCl 119; NaHCO_3_ 24; KCl 4.7; MgSO_4_ 1.17; KH_2_PO_4_ 1.18; CaCl_2_ 1.6; glucose 5).

The first segment of the MCA immediately proximal to the circle of Willis, that was branch-free, was dissected out and placed in cold PSS (4°C). The vessel was mounted on two glass micropipettes (diameter, 125–150 µm) in a small vessel arteriograph (model CH/1/AU/SH, Living Systems Instrumentation, Burlington, VT, USA) and secured with silk thread (diameter, 20 µm, 2–0, 48 mm, 2540, Ethicon, Issy-les-Moulineaux, France). The vessel was then pressurized with PSS in an organ bath containing extraluminal PSS at 37°C at an intraluminal pressure equal to 60 mmHg in WKY and SHR+TELMI and 100 mmHg in SHR (*i.e.* 60–70% of MAP) in no-flow condition. The vessels were superfused with PSS gassed with 5% CO_2_/95% O_2_. Changes in ID were measured using a video dimension analyser (V94, Living Systems Instrumentation, Burlington, VT, USA); signals were digitized and stored with WinDaq data acquisition software (Dataq Instruments, Akron, OH, USA).

All MCA were pressurized throughout the experiment. Following initial pressurisation and the increase of wall stretch, ID immediately rose, then decreased and stabilized over the next 60 minutes (baseline ID). Viability was next confirmed by responses to potassium chloride (KCl, 40 mM), then serotonin (5-HT, 1 µM) added for 5 minutes to the arteriograph bath. Arteries that contracted less than 20% were discarded (15–20% of exclusion, no difference between groups, results not shown).

### Experimental protocols

Myogenic and stress strain curves were built in the same series of MCA (n = 9–13 rats/group). For myogenic curves, intraluminal pressure was reduced to 20 mmHg and then increased in 20 mmHg increments up to 200 mmHg in a stepwise manner. Vessels were allowed to stabilize at each pressure for 3 min. Intraluminal pressure was then reduced back to the initial value for 5 minutes. PSS was then replaced with calcium-free/EDTA PSS (same composition as PSS with CaCl_2_ omitted and with 20 mM EDTA added). Ten minutes after replacement of PSS, the intraluminal pressure increments were repeated in Ca^2+^-free conditions to determine passive vascular characteristics.

Myogenic tone was evaluated for each pressure step from the change in ID following smooth muscle cell inactivation, *i.e.* from the increase in ID (1−(ID_EDTA_/ID)×100).

Then pressure curves and stress strain curves were built and elastic modulus was calculated. Stress (σ) was calculated as: σ = 0.001334×pressure×ID/(2×wall thickness) and strain (ε) as: ε = (ID-ID_O_)/ID_O_ where ID_O_ is the internal diameter at the lower pressure step (20 mmHg). The tangential elastic modulus (β), an index of parietal distensibility, was defined by the following equation: σ = σ_O_.e^βε^ where σ_O_ is the stress at the lower pressure step (20 mmHg).

In another series of MCA (n = 8–9 rats/group), cumulative concentration responses to AngII were performed (10^−12^ to 10^−6 ^M). Responses to AngII were expressed as E_max_ (maximum increase in ID, %) and EC_50%_ (concentration in M that evokes 50% of E_max_).

#### Substances used

TELMI was provided by Boehringer Ingelheim Pharma GmbH & Co. KG (Ingelheim am Rhein, Germany). For chronic treatment, telmisartan solutions were prepared twice a week, according to Heinemann *et al.,* 1997. AngII, KCl, 5-HT and EDTA were purchased from Sigma Chemical Company (St Louis, MO, USA), and sodium pentobarbitone from Sanofi-Aventis (Libourne, France). For application in the bath, all drugs were dissolved in PSS.

### Statistical analysis

Statistical analyses were performed using GraphPad Prism version 5 for Windows, (GraphPad Software, La Jolla California USA, www.graphpad.com). Results are expressed as means ± s.e.m. Significant differences between the groups were determined by a one-way ANOVA followed by a post-hoc Tukey’s multiple comparisons test. The null hypothesis was rejected at p<0.05.

## Results

**Figure 1:**
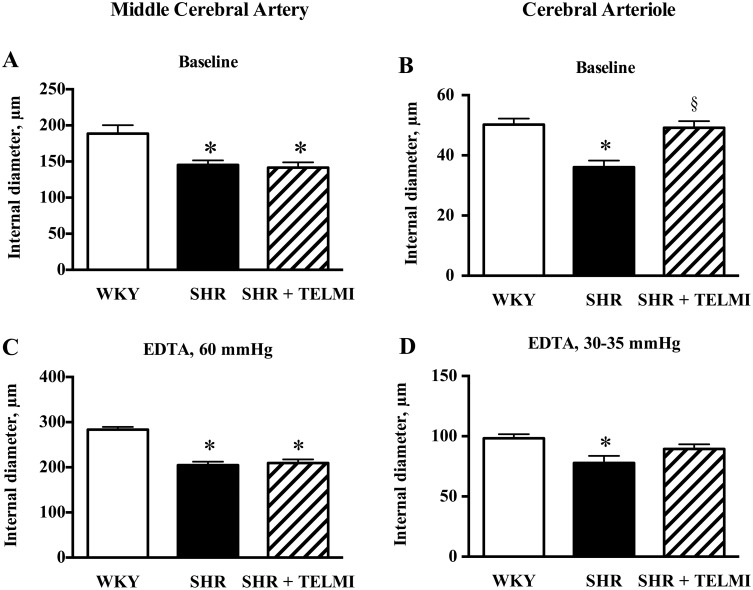
Internal diameters at baseline (panels A and B) and after inactivation with EDTA (panels C and D) of middle cerebral arteries (present study, panel A, n = 17–21; and panel C, n = 9–13) and of cerebral arterioles (previous published study, panel B, n = 11–15; panel D, n = 9–12) from WKY, SHR and SHR treated for 10 days with telmisartan (SHR + TELMI, 2 mg/kg per day). One-way ANOVA (panel A, p = 0.0004; panel B, p<0.0001; panel C, p<0.0001; panel D, p = 0.0152) + Tukey’s multiple comparisons test: *p<0.05 *vs* WKY; § p<0.05 *vs* SHR.

MAP was 46% higher in SHR (168±3 mmHg) than in WKY (115±2); TELMI lowered MAP by 24% (129±3, One-way ANOVA p<0.0001, Tukey’s multiple comparisons tests p<0.05 SHR vs WKY, TELMI vs SHR, WKY vs TELMI). Baseline active (Figure 1A) and passive (Figure 1C) ID were 23 and 28% lower in SHR than in WKY respectively; TELMI had no effect, contrary to the results obtained in pial arterioles (Foulquier *et al*., 2012) (Figure 1B/1D).

TELMI abolished vasoconstriction in response to AngII (E_max_ SHR −21±3, WKY −20±2, SHR + TELMI −7±2%; One-way ANOVA p = 0.006, Tukey’s multiple comparisons test, p<0.05 SHR + TELMI vs WKY and SHR; EC_50%_ SHR 1±1.10^−10^, WKY 2±1.10^−9^, SHR + TELMI 1±1.10^−10 ^M, n = 7–9 per group).

**Figure 2:**
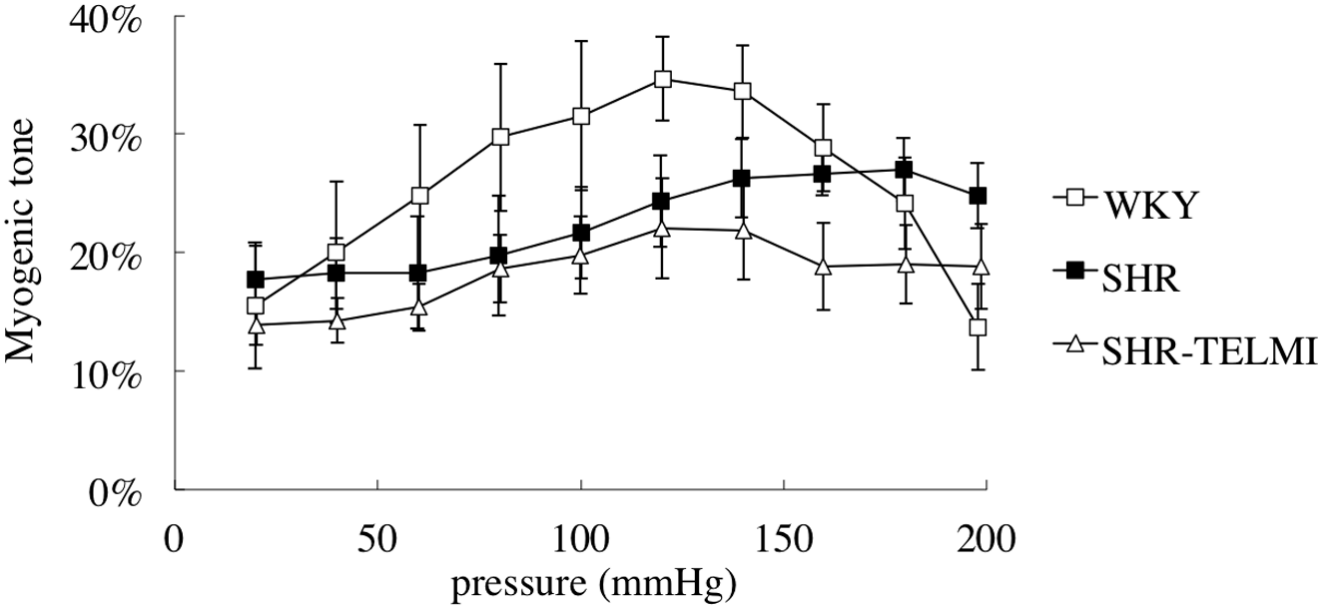
Myogenic pressure curves of middle cerebral arteries from WKY (n = 9, open squares), SHR (n = 6, full squares) and SHR treated with telmisartan (SHR + TELMI, 2 mg/kg per day, n = 7, open triangles).

Neither hypertension (SHR *vs* WKY) nor TELMI (SHR + TELMI *vs* SHR) had any significant effect on the level of myogenic tone (maximal myogenic tone SHR 27±3%, WKY 35±4%, SHR + TELMI 22±4%; One-way ANOVA p = 0.006, Tukey’s multiple comparisons tests p>0.05) but pressure myogenic curves were shifted to higher pressure values in SHR (maximum values obtained at 180 mmHg) compared to WKY (maximum values at 120 mmHg). TELMI shifted the pressure myogenic curve to lower pressure values than SHR (maximum values at 140 mmHg, Figure 2).

**Figure 3:**
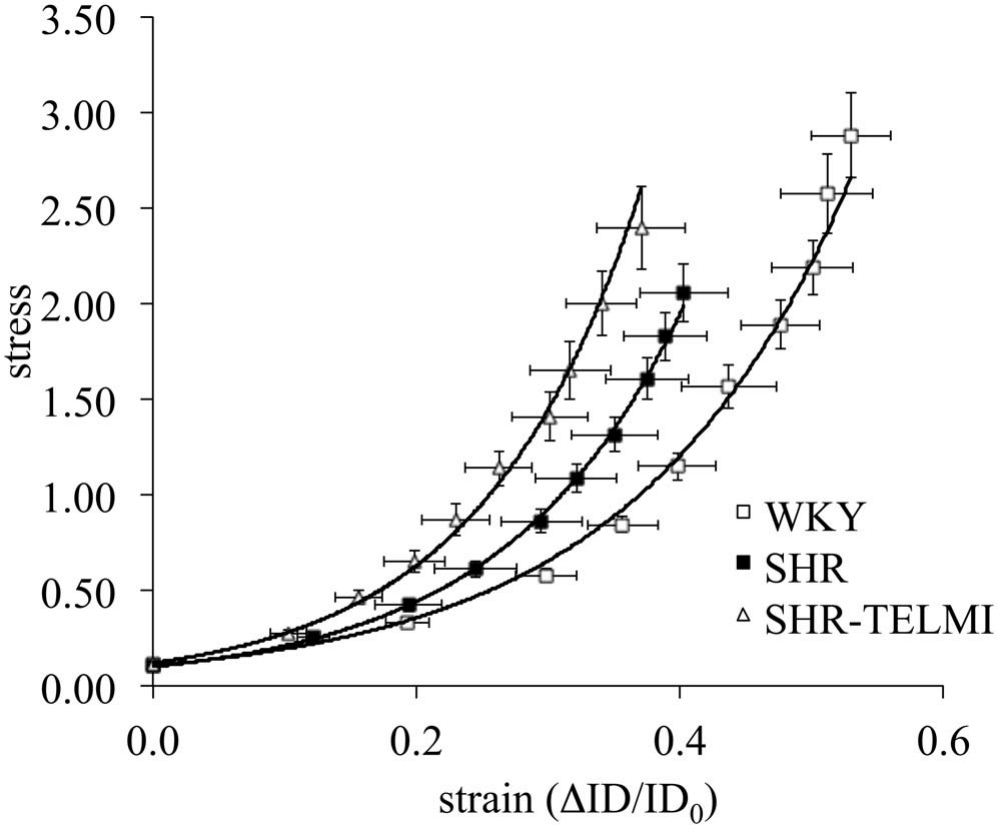
Stress–strain relationships in deactivated middle cerebral arteries of WKY (n = 11, open squares), SHR (n = 13, full squares) and SHR treated with telmisartan (SHR + TELMI, 2 mg/kg per day, n = 9, open triangles). The slope of each stress-strain curve corresponds to the elastic modulus of the vessel studied. Elastic moduli: WKY 6.2±0.4; SHR 7.7±0.7; SHR + TELMI 8.4±0.7; One-way ANOVA P = 0.045, Tukey’s multiple comparisons test, p<0.05 SHR + TELMI vs WKY).

Stress strain curves were shifted to the left in SHR and SHR + TELMI compared to WKY (Figure 3), showing a tendency for decreased elastic properties. The ANOVA identified differences for the elastic moduli between the groups but TELMI has no effect *versus* SHR (elastic modulus WKY 6.2±0.4; SHR 7.7±0.7; SHR + TELMI 8.4±0.7; One-way ANOVA p = 0.045, Tukey’s multiple comparisons test, p<0.05 SHR+ TELMI vs WKY).

## Discussion

Narrowing of ID of MCA and pial arterioles in SHR is mainly due to structural remodeling (decrease in passive ID) [13], with no changes in vasomotion nor myogenic tone (present results) [2], [3], [10]. This contributes to the increase in cerebrovascular resistance and to the rightward shift of the limits of cerebral blood flow autoregulation, protecting the brain against higher blood pressure during hypertension. At the onset of an antihypertensive treatment, it is beneficial to induce early reversal of remodeling.

Here, 10-day treatment with TELMI, while efficient enough to decrease blood pressure and to abolish the response to AngII, did not reverse arterial remodeling of MCA in young adult SHR. These results are in opposition to those already published for pial arterioles [10] where we showed that 10-day treatment at 2 mg/kg per day reverses the narrowing of pial arteriolar ID in SHR, *via* both reversal of structural remodeling and inhibition of the AngII vasoconstrictor response [10]. In MCA, structural remodelling, assessed by passive ID, was not affected by TELMI treatment, suggesting that MCA require longer duration of blockade of the renin angiotensin system than arterioles to reverse structural remodeling. Therefore, the ability of a treatment with TELMI to reverse the hypertension-induced remodeling at short term may be dependent of the vasculature size, starting from micro- to macro-circulation. Larger vessels’ wall obviously contains more material than smaller ones and would thus require more time for changes in composition and/or in the interactions between its components to be measurable. This should be confirmed by further experiments to solve some of our study limitations: (i) the lack of timepoints that narrow our interpretations, and (ii) the fact that studying both cerebral micro- and macro-circulation within the same animal would have been more relevant, although technically difficult.

Despite the abolishment of the vasoconstrictor responses to AngII observed in MCA following TELMI treatment, we did not observed any change in baseline ID of MCA, suggesting that, contrary to what happens in pial arterioles, basal AngII-related vasoconstrictor tone in MCA is certainly not high enough to influence baseline ID. Taken together, these results reflect important differences between the cerebral arterial and the cerebral arteriolar responses to blockade of the AngII type 1 receptor (AT1R).

In addition to remodeling, two other factors are involved in cerebral autoregulation, vessel wall distensibility and myogenic responses to changes in perfusion pressure. One of the main differences between arteries and arterioles is that arteriolar wall distensibility increases with hypertension, in relation with accumulation of more elastic components inside the arteriolar wall [1]–[3], [14], whereas no change, or even a tendency to decrease distensibility occurs in larger arteries (present study) [15], [16]. This suggests that the relative proportion of stiff and elastic components within the arterial wall differs from the arteriolar wall. This difference could also contribute to the lack of change in structural remodeling of MCA following short-term TELMI treatment when compared to pial arterioles. However, this would need further exploration as TELMI did not change wall distensibility of cerebral arterioles [10] nor that of MCA (present study).

A decrease in distensibility may lead to deleterious responses such as lower vasodilation capacities following stroke [17]. However, as cerebral circulation is regulated at different levels [11], [12], arterioles downstream of the MCA are able to compensate and adapt their capacities for autoregulation. For example, small arterioles start to dilate for greater changes in blood perfusion pressure than large cerebral arteries [18], [19]. Furthermore, the increased distensibility of cerebral arterioles is interpreted as a protective mechanism to adapt capacities of autoregulation [1], [20], [21].

Myogenic response to changes in perfusion pressure is also involved in autoregulation [22], [23]. MCA myogenic curves - even if not associated with any change in amplitude of myogenic tone - are shifted to the right in SHR. This is probably an adaptative mechanism to maintain dilatory capacities for higher blood pressure values. Thus, it likely participates to the rightward shift of cerebral blood flow autoregulation in order to protect the brain from higher blood pressure values. While TELMI did not change the level of myogenic tone, it shifted the pressure myogenic curve to lower pressure values than SHR, in relation to the decrease in blood pressure it induced. This may allow cerebral circulation to early adapt towards lower blood pressure values.

In conclusion, short-term treatment with TELMI had no effect on structural remodeling and did not restore baseline ID of MCA from SHR, on the contrary to what happens in cerebral arterioles, but allowed myogenic tone to adapt towards lower pressure values. This may be the only early beneficial effect on large cerebral input arteries, susceptible to protect the brain against the early decrease in pressure at the onset of treatment.

While dealing with anti-hypertensive therapies in post-stroke patients with elevated blood pressure, clinicians may consider telmisartan as a valuable option. Indeed, despite its lack of impact on arterial remodeling, telmisartan reverses arteriolar remodeling [10] and restores the arterial myogenic curve. Future research should evaluate in a time-dependent manner whether this differential remodeling between macro- and micro-circulation occurs in humans as well. This will provide more insights regarding the beneficial effects of ARBs in addition to their blood pressure lowering actions and will help clinicians to establish guidelines for the early management of cerebrovascular diseases including stroke. Finally, compared to the clinical situation, the animals used were young adults. It would be thus interesting to evaluate the impact of such short term treatment with TELMI in older animals in which the vascular remodeling due to hypertension is established since longer time.
